# Rare Anaphylactic Reaction to Acamprosate in a Young Alcoholic

**DOI:** 10.7759/cureus.6210

**Published:** 2019-11-20

**Authors:** Asha Patnaik, Barjinder S Buttar, Basma Ataallah, Vikas Kumar

**Affiliations:** 1 Rheumatology, Stony Brook University, Stony Brook, USA; 2 Internal Medicine, Northwell Health Mather Hospital, Port Jefferson, USA; 3 Internal Medicine, Zucker School of Medicine at Mather, New York, USA; 4 Internal Medicine, Donald and Barbara Zucker School of Medicine at Hofstra / Northwell, Port Jefferson, USA

**Keywords:** allergy, immunology, alcoholism, anaphylaxis, preventive medicine

## Abstract

Alcoholism is a worldwide health issue that affects people from all walks of life. Effective alcohol abuse treatment programs, which combine cognitive behavioral therapy with medications, are necessary to provide the appropriate care that patients need. Treatment options include Naltrexone, Acamprosate, or Disulfiram. Due to poor compliance, Disulfiram is used as a second line agent. These medications play an integral role in treating alcohol abuse. Healthcare providers must be aware of their side effect profiles. This includes a rare and fatal anaphylactic reaction to Acamprosate which is not well documented in the current literature.

## Introduction

Alcoholism truly is a worldwide health issue that shows no signs of slowing down. In the United States, from 2006 to 2010, alcohol killed 88,000 Americans and from 2007 to 2017 the number of alcohol-related deaths in the United States had increased by 35% [1]. Due to the widespread prevalence of this disease, it is necessary for healthcare providers to have a thorough understanding of the treatment options available for managing patients struggling with alcoholism. This includes medications, their mechanisms of action, and their side effect profiles. When treating patients, it is important to keep in mind that medications do not treat the behavioral issues associated with addiction, and medications must be given in combination with cognitive behavioral therapy. The three medications that are both safe and effective for treating alcoholism include Disulfiram, Naltrexone, and Acamprosate.

## Case presentation

**Figure 1:**
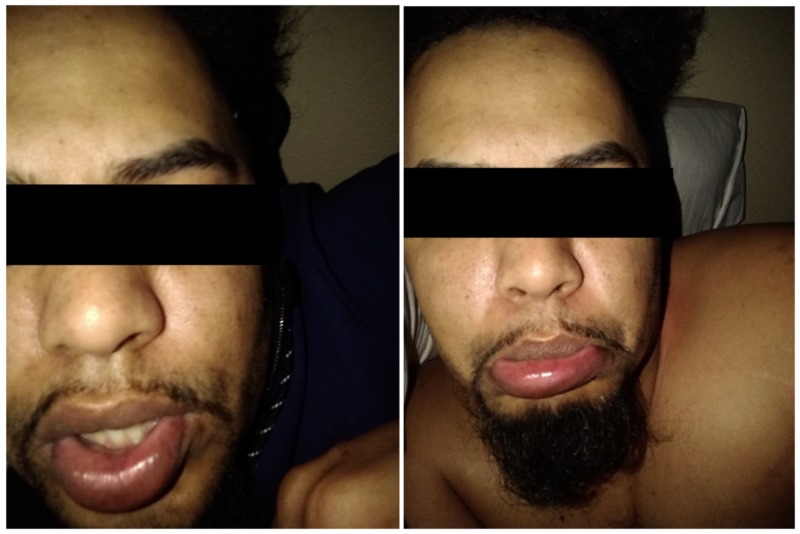
Significant urticaria and angioedema of the bottom lip and face after taking Acamprosate

This case report will focus on a 32-year-old homeless male with a long history of alcohol abuse with multiple relapse episodes. He states that he is currently abstinent and had recently been started on Acamprosate by his primary care physician. He is living in a shelter with his girlfriend who had just recovered from an infection of her bilateral axilla. The patient stated that he used his girlfriend's deodorant which led to the development of large, painful boils under his right axilla and groin simultaneously. After taking a warm bath he self-lanced both boils and later that day he began to experience fatigue, muscle aches, pains, and fevers. Later that night he took Acamprosate with an energy drink. Shortly after taking it he developed severe urticaria and pruritis. He went to bed and the next morning he developed significant angioedema of the bottom lip as shown in Figure 1A, 1B below.

**Figure 2:**
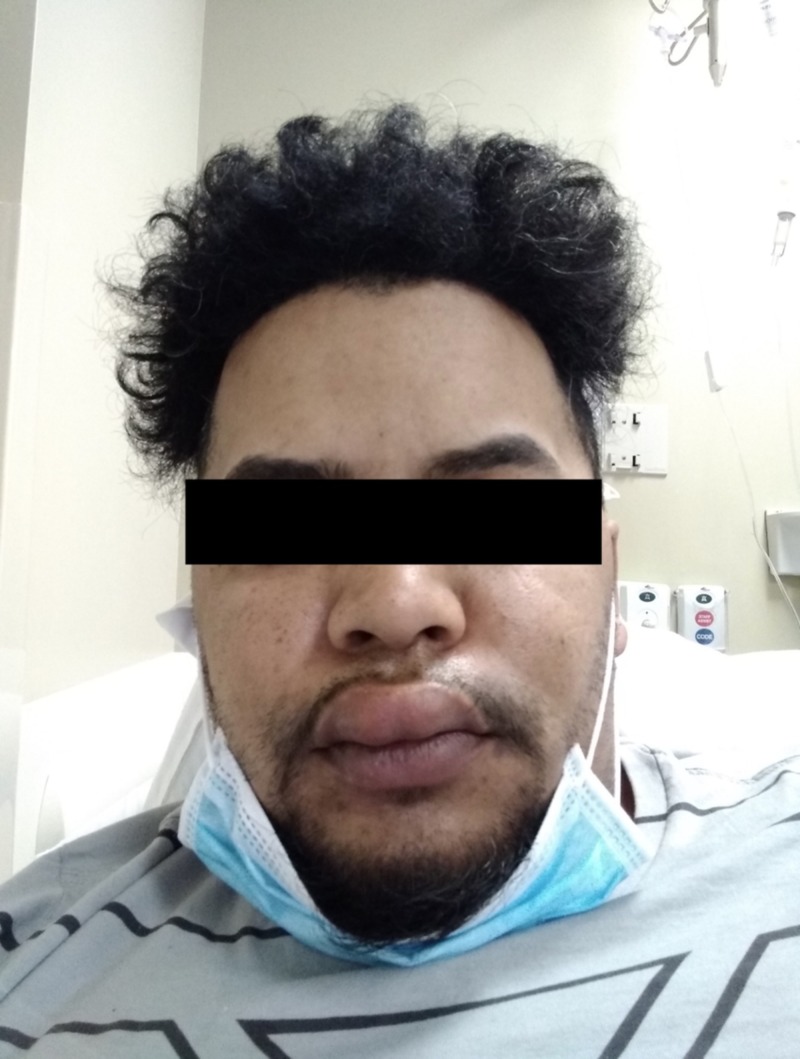
Progression of angioedema to the upper lip

The patient went to an urgent care center, was treated with Prednisone, and had a nasal swab for influenza which was positive. He was treated with Tamiflu and sent home. He went on to develop significant angioedema of the upper lip as seen in Figure 2 below.

Due to the persistent and severe nature of the symptoms the patient decided to go to the emergency room. Acamprosate was immediately discontinued and the patient was informed to never take this medication again. He was treated with steroids and Benadryl with gradual improvement of symptoms until complete resolution.

## Discussion

Disulfiram, commercially known as Antabuse, is an alcohol aversive agent that is effective in treating alcohol dependence. Its mechanism of action is not well understood but it likely involves an increase in the body’s sensitivity to ethanol by inhibition of the enzyme acetaldehyde dehydrogenase (ALDH) [2]. Studies have shown that the side effect profile of Disulfiram is minimal and generally safe to use when treating alcohol use or dependence. It is, however, important to inform patients of the potentially severe and fatal disulfiram reaction which occurs when drinking alcohol while taking the medication. Patients can present with severe flushing, hypotension and tachycardia. Minor common side effects include skin rash, halitosis, and fatigue. Rare side effects include neurological, psychiatric, hepatic, and cardiac events [3].

Naltrexone is a pure opioid antagonist with activity at multiple opioid and non-opioid human receptors. It is licensed to be used as an aid to prevent relapse in alcohol use disorders and opioid addiction after withdrawal. It is contra-indicated in patients who are actively using opioids due to the high risk of overdosing on opioids or adverse events related to rapid opioid withdrawal [4]. Studies have confirmed that oral naltrexone, when used in licensed indications, does not appear to increase the risk of serious adverse events over placebo. Minor common side effects include nausea, vomiting, abdominal pain, decreased appetite, dizziness, lethargy, headaches, and sleep disorders [4].

In addition to Disulfiram and Naltrexone, Acamprosate is licensed for prevention of relapse in alcohol dependence as well. Studies have shown that it is effective, safe, and tolerable for the majority of patients [5]. Acamprosate is well known as an anti-craving drug that diminishes withdrawal symptoms including hyperarousal, anxiety, and insomnia which may cause an individual to crave alcohol for relieve. Its exact mechanism of action, however, is not well understood. It is believed to modulate N-methyl-d-aspartic acid receptor (NMDA) transmission and may have indirect effects on γ-aminobutyric acid (GABA) type A receptor transmission. It is also known to decrease brain glutamate and increase β-endorphins [5]. The most common side effects reported include diarrhea within the first four weeks of treatment, abdominal pain, constipation, nausea, vomiting, headache, pruritis, and vertigo. It is also excreted by the kidneys and contraindicated in patients with renal impairment [6]. Although rare, and not well documented in the current literature, severe and potentially fatal hypersensitivity reactions to Acamprosate are present. These patients can present with widespread urticaria and angioedema progressing to fatal anaphylaxis with swelling of the lips, tongue, and throat causing respiratory compromise. Identifying these patients immediately, stopping the medication, and treating them with antihistamines, steroids, and epinephrine if needed is essential.

## Conclusions

Hypersensitivity reactions to Acamprosate are rare and not well documented in the literature but do occur. Patients dealing with alcohol abuse tend to have complicated histories with multiple co-morbid conditions which can make it difficult to identify the source of symptoms. Missing this diagnosis will have dangerous consequences and can lead to death. Healthcare providers using this medication need to be well educated when it comes to this serious reaction.
